# DHA Sensor GPR120 in Host Defense Exhibits the Dual Characteristics of Regulating Dendritic Cell Function and Skewing the Balance of Th17/Tregs

**DOI:** 10.7150/ijbs.39551

**Published:** 2020-01-01

**Authors:** Caiquan Zhao, Jinxiu Zhou, Yanqing Meng, Niu Shi, Xiao Wang, Ming Zhou, Guangpeng Li, Yang Yang

**Affiliations:** 1 The State Key Laboratory of Reproductive Regulation and Breeding of Grassland Livestock, School of Life Sciences, Inner Mongolia University, Hohhot, 010070, China; 2 The State Key Laboratory of Agricultural Microbiology, College of Veterinary Medicine, Huazhong Agricultural University, Wuhan, 430070, China; 3 Inner Mongolia People's Hospital, Hohhot, Inner Mongolia, CN 010017

**Keywords:** DHA, GPR120, dendritic cell, Th17/Tregs, rabies virus, Japanese encephalitis virus

## Abstract

In addition to functioning as an antioxidant, anti-inflammatory and age-defying cellular component, DHA impacts the immune system by facilitating the pathogen invasion. The mechanism through which DHA regulates immune suppression remains obscure. In our study, we postulated that DHA might interact with GPR120 to shape the dendritic cell (DC) differentiation and subsequently drive T cell proliferation during the virus infection. *In vitro*, the proportion of costimulatory molecules and HLA-DR on DC that generated from exogenous and endogenous (*fad3b* expression) DHA supplemented mice were significantly lower than wild-type mice. Given the importance of FAs, DHA is not only a critical cellular constituent but also a cell signaling molecule and FA deficiency reduces DC generation; we used GPR120^-/-^ mice to determine whether DHA receptor deficiency disorders DC maturation processing. Novelty, the expression of GPR120 on DC from wild-type (WT) mice was inversely related to DC activation and DC from the GPR120^-/-^ mice maintained a spontaneous maturation status. *In vivo*, both the excessive activation of GPR120 by DHA and the deletion of GPR120 effectively skewed the balance of Th17/Tregs and reduced the production of VNA and protection of vaccination. Overall, our results revealed a mechanism that the GPR120 self-regulation plays a crucial role in sensing DHA variation, which provides a new prospect for therapeutic manipulation in autoimmune diseases and the design of a vaccine adjuvant.

## Introduction

Vaccination is the main means of preventing and controlling the spread of many infectious diseases 1, 2. For some infections, such as measles, polio, rabies and Japanese encephalitis (JE), vaccination is the most effective method for controlling and preventing infections 3-7. If vaccination fails, the course of the disease can be irreversible, leading to irremediable consequences. Moreover, immune deficiencies such as autoimmune disease or immunosuppression could induce a vaccination failure or some patients suffering a disease of the immune system may contraindicate the use of some vaccines 8-10. A better knowledge of the mechanisms involved in immune response induction by vaccines could greatly facilitate vaccine development.

DC are the professional antigen present cells (APCs) that bridge innate immune responses and adaptive immune responses against pathogens and tumors 11. In the state of immune stability, the DC distributed in peripheral lymphoid tissues is in the immature state that expresses a high level of pattern recognition receptors (PRRs), which leads to the production of various cytokines, chemokines and interferon 12. The DC recognizes and responds to an infection and triggers the maturation process to recognize and present antigens and shapes the regulatory phenotype of T cells 13. Several metabolic switches or genes have been identified that induce tolerogenic or autoimmune DC biology 14. Therefore, it is necessary to understand the cellular biochemistry of DC, which will facilitate the design of vaccine immunity procedures and prevent autoimmune diseases from inhibiting the effects of the vaccine.

Fatty acids (FAs) act as an essential element of cellular metabolism. FA metabolism alters the proliferation and activation of the myeloid and lymphoid lineages of blood cells 15. Both n-3 polyunsaturated fatty acids (n-3 PUFAs) deficiency and supplementation have crucial effects on the development and function of these cells. For example, the n-3 PUFAs in fish oil exert suppression of some symptoms associated with acute and chronic inflammation 16. Inflammatory responses involve varies of cells, and a significant body of literature shows that n-3 PUFAs suppress NK cells 17, DC 18, macrophages 19, 20 and T cell functions 21, 22, as measured by cytokine secretion and cell proliferation. Furthermore, the anti-inflammatory properties of PUFAs may be beneficial against some chronic inflammatory illnesses, but dietary n-3 PUFAs can impair host resistance to intracellular pathogens. For instance, supplementation with n-3 PUFAs has been shown to increase host susceptibility to *Mycobacterium tuberculosis* in the lung 23, *Listeria monocytogenes* in the liver 24, *Salmonella typhimurium* in the spleen 25, and *Influenza* in the lung 26. Similarly, *fat-1* mice (which endogenously produce n-3 PUFAs) also show increased susceptibility to *Mycobacterium tuberculosis* 20. Compared with high n-3 PUFAs supplementation, low fish oil intake increases cell-mediated immunity and enhances certain immune functions *in vivo* and *in vitro* 27, 28. Furthermore, low n-3 PUFAs intake enhanced lymphocyte proliferation and the secretion of TNF-α and IL-1β 29. However, dependent on a critical review, they performed a statistical analysis over the past decades, which demonstrated a nearly equal number of published studies that report adverse effects of n-3 PUFAs on fighting infection to those that show a beneficial effect 25. To generally infer the effect of n-3 PUFAs on the immune response from these results was inconclusive. A major limitation of n-3 PUFAs studies was a lack of specific targets and mechanisms of n-3 PUFAs at the molecular level.

G protein-coupled receptors (GPCRs) are essential signaling modulators for regulating cellular functions. Approximately hundreds of GPCRs are still defined as “orphan” receptors without known ligands 30. Among these, GPR41 and GPR43 serve as receptors of short-chain fatty acids 31, and GPR40 and GPR120 receptors recognize long-chain fatty acids 32. Especially, GPR120 is a functional n-3 PUFAs receptor, which mediates potent inflammatory suppression by blocking cellar immune functions and cytokine or chemokine secretion 19, 33. Furthermore, GPR120 is a negative regulator of inflammatory processing in macrophages 19. Several studies have reported that the lack of some generally negative regulators of immune activation caused inflammation or spontaneous maturation of DC, and improper DC activation probably initiates an immune deficiency 34-37. The spontaneous maturation of DC leads to a tolerogenic type of immune response, which is not sensitive to stimuli 38. Autoimmunity diseases may also promote infections, and viruses are protected by autoimmunity 39-41. Although, GPR120 has been implicated in DHA dependent inflammatory regulation, its function in innate immunity has never been reported. DC exert diverse functions in immune regulation and are distinguished by various surface and intracellular markers. Thus, the expression of GPR120 on DC might play a vital role in regulating cellular functions.

We investigated the role of GPR120 and DHA in shaping DC and T cells with *in vivo* GPR120^-/-^ mice and *fad3b* mice models and *in vitro* treatment of DC with GPR120 agonists and different fatty acids. Our *in vitro* analysis revealed that FAs metabolism was enhanced with DC maturation and DHA through GPR120 to regulate DC maturation. Furthermore, the expression of GPR120 was markedly reduced after DC maturation, and mature DC were unaffected by DHA. *In vivo*, we revealed that both the DHA supplemented mice and DHA receptor-deficient (GPR120^-/-^) mice displayed a further increased susceptibility to viral infection. Our results demonstrated that DHA-GPR120 interaction is critical for maintaining the immature state of DC and is required for host defense against viral infection.

## Results

### FA metabolites are accumulated and required during DC differentiation and activation

**Figure 1:**
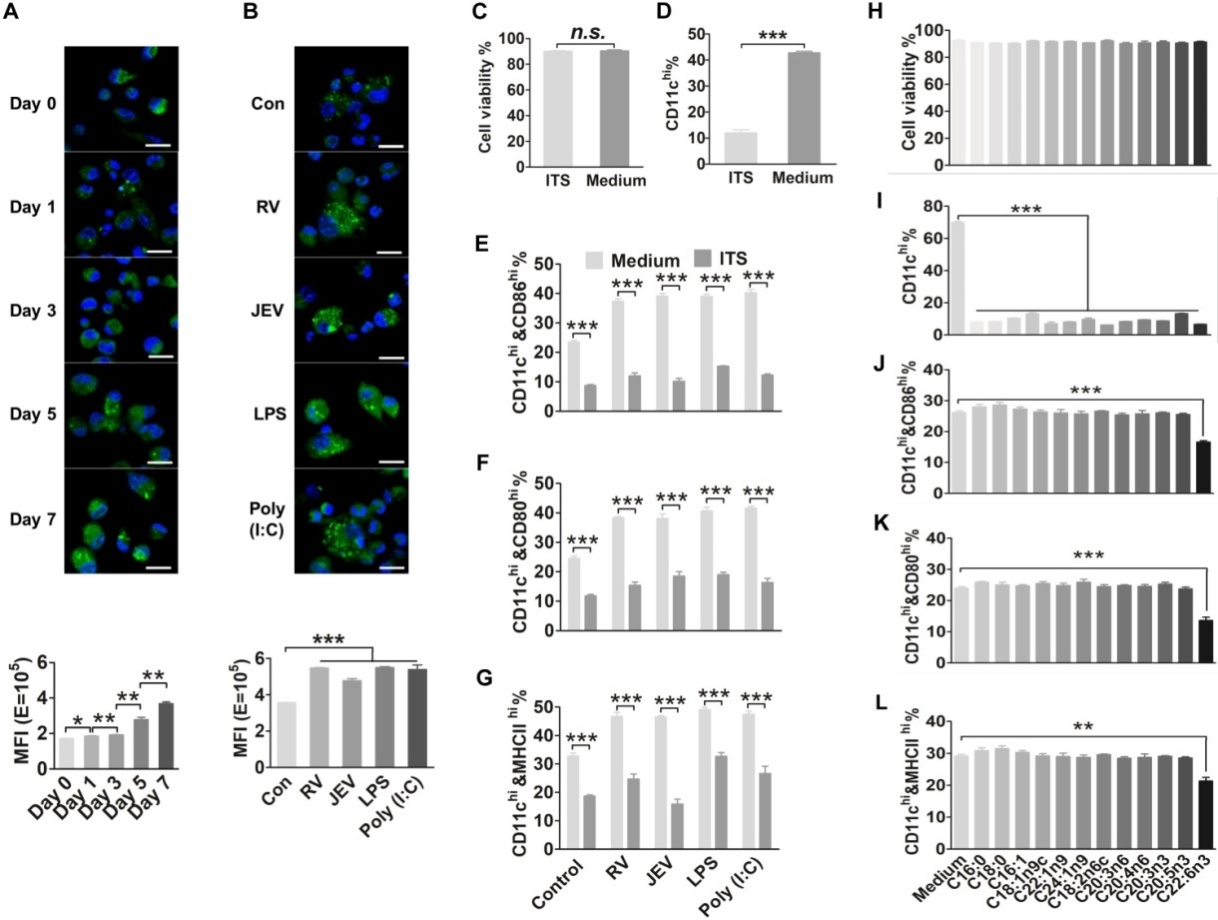
** FA metabolism is required for DC generation and activation.** (A) Quantification and visualization of intracellular neutral lipids in immature DC. Bone marrow cells were cultured in 20 ng/ml GM-CSF (granulocyte-macrophage colony-stimulating factor, GM-CSF) and collected for staining with Bodipy 493/503 droplets (green) and DAPI (blue) at day 0, 1, 3, 5, and 7, and analyzed with confocal microscopy (top) and FACS (bottom). The scale bar of each image is 50 μm. (B) Quantification and visualization of intracellular neutral lipids in mature DC. DC were stimulated with RV, JEV, LPS, or Poly (I:C) for 24 h and collected for staining with Bodipy 493/503 droplets (green) and DAPI (blue) and analyzed with confocal microscopy (top) and FACS (fluorescence-activated cell sorter, FACS) (bottom). The scale bar of each image is 50 μm. (C) Analysis of DC viability in an FAs-free medium or complete medium using trypan blue. The n.s. stands for no significance. (D) Flow-cytometric analysis of the proportion of CD11c on BMDC in an FAs-free medium or complete medium. (E-G) Flow-cytometric analysis of the proportion of CD86, CD80 and MHCII in CD11c-positive cells after treatment with RV, JEV, LPS and Poly (I:C) for 24 h in an FA-free medium or complete medium. (H) Analysis of DC viability in a series of indicated FAs under complete medium using trypan blue. (I) Flow-cytometric analysis of the proportion of CD11c on BMDC in a series of indicated FAs under complete medium. (J-L) Flow-cytometric analysis of the proportion of CD86, CD80, and MHCII in CD11c-positive cells after treatment with RV, JEV, LPS and Poly (I:C) for 24 h in a series of indicated FAs under complete medium.

To date, several studies have reported that FAs can work as an endogenous ligand signal to modulate inflammatory and immune response 19, 42. In the present study, the lipid content of DC was staining with the fluorescent neutral lipid dye. We found that the content of FAs in single-cell or total DC was increased dramatically during the generation of bone marrow-derived dendritic cell (bmDC) (Fig. 1A). Additionally, DC treated with vaccine strains of viral JEV and RV, LPS or Poly (I:C) also enhanced FA content after 24 hours of stimulation (Fig. 1B). We inferred that some intracellular FAs might serve as a cellular signal peptide, which may influence DC maturation. The bmDC were collected to evaluate a total of 37 kinds of FAs by GC/MS. In regard to the FA profiles, three saturated FAs (C14:0, C16:0, and C18:0) and seven unsaturated FAs (C18:1n9c, C18:2n6c, C20:3n6, C20:3n3, C20:4n6, C20:5n3, C22:6n3) were significantly increased during DC generation and activation (Fig. S1 A-D).

To determine whether FAs alter the generation and maturation of DC and in turn affect anti-viral immune responses, bone marrow (BM) cells were cultured under FA-free conditions for seven days in the presence of GM-CSF to measure the expression of CD11c on cell surfaces and cell viability. There was no significant difference in cell viability between the FA-free and complete conditions (Fig. 1C). However, the FA-free medium resulted in an approximately 30% reduction of CD11c^+^ cells in total cells (Fig. 1D) and more than 15% reduction of CD86^+^, CD80^+^ or MHCⅡ^+^ cells in CD11c^+^ cells comparing to complete medium (Fig. 1E-G). Furthermore, it is difficult to activate DC under FA-free conditions. A series of FAs such as C16:0, C18:0, C16:1, C18:1n9c, C22:1n9, C24:1n9, C18:2n6c, C20:3n6, C20:3n3, C20:4n6, C20:5n3 and C22:6n3 were respectively supplemented into complete medium or FA-free medium. There were no significant differences in cell viability between the complete and FA supplemented medium (Fig. 1H). Under FA-free conditions, none of the single FAs alone could generate a similar proportion of CD11c^+^ cells compared with the complete medium (Fig. 1I). However, only DHA supplementation suppressed CD86, CD80, and MHCII expression in DC under complete medium (Fig. 1J-L). We excluded the possibility that FAs are not only an essential nutritional component but also a critical signaling molecule in DC generation and maturation processes.

### DHA supplementation suppresses DC maturation and enhances the Tregs that reduce the protection of vaccinations

**Figure 2:**
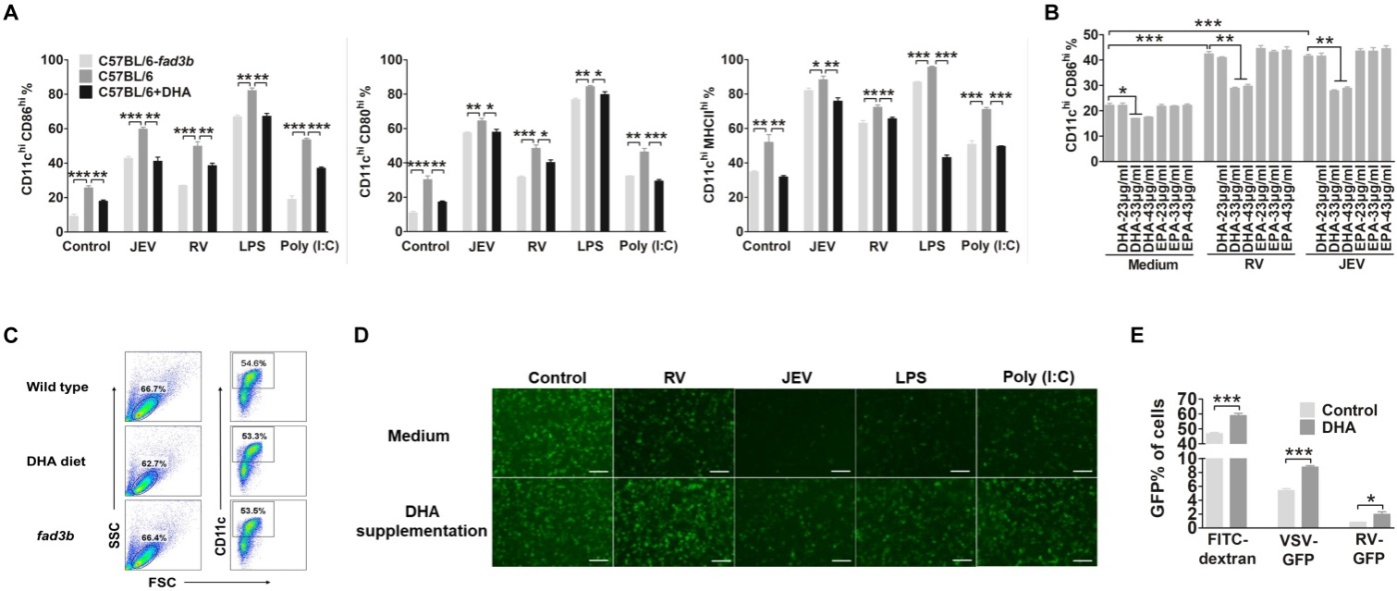
** DHA renders a DC resting state**. (A) Flow-cytometric analysis of the proportion of CD86, CD80, and MHCII in CD11c positive cells after treatment with RV, JEV, LPS, and Poly (I:C) for 24 h in WT C57BL/6 mouse derived bmDC in DHA supplementation medium or *fad3b* mouse derived bmDC in complete medium. (B) Flow-cytometric analysis of the proportion of CD86 in CD11c-positive cells after treatment with RV, JEV, LPS, and Poly (I:C) for 24 h in WT C57BL/6 mouse derived bmDC with DHA or EPA supplementation at different indicated concentrations. (C) Flow-cytometric analysis of the proportion of the bone marrow derived cell and CD11c positive from wild-type, DHA diet and *fad3b* mice. (D) DC were stimulated with RV, JEV, LPS, or Poly (I:C) and the supernatant was used to test type I IFN with VSV-GFP. The type I IFN production was inversely correlated with GFP expression. Images are representative of two independent experiments, and the scale bar of each image is 50 μm. (E) Flow-cytometric analysis of DC macropinocytosis with FITC-dextran, VSV-GFP, or RV-GFP in complete medium or DHA supplementation medium.

To determine the effect of DHA supplementation on DC maturation and anti-viral responses, we evaluated the costimulatory molecule and HLA-DR on DC *in vitro*. The bmDC were treated with an extra 33 μg/ml of DHA for 12 hours before priming with RV, JEV, LPS or Poly (I:C). The expression of CD86, CD80, and MHCII on DHA treated DC were significantly decreased. To simulate the rise of endogenous FAs in DC, DC derived from the bone marrow of *fad3b* mice, which overproduces endogenous n-3 PUFAs (Fig. S2), also inhibit DC activation (Fig. 2A). Furthermore, DHA impaired the maturation of DC in a dose-dependent manner and the concentration of DHA less than 23 μg/ml hardly inhibited the maturation of DC (Fig. 2B). Besides, the amount of bone marrow derived cells and CD11c+ cells that generated from exogenous and endogenous DHA supplemented mice bone marrow was similar to the wild-type (WT) mice (Figure 2C). The DHA treatment also decreased the IFN secretion level (Fig. 2D). Next, we examined the macropinocytosis of DC by incubating with FITC-dextran, VSV-GFP or RV-GFP. DHA treated DC showed higher uptake of FITC-dextran and virus (Fig. 2E). Together, these data demonstrate that DHA renders DC in an immature state even following stimulation with a virus, LPS or Poly (I:C).

**Figure 3:**
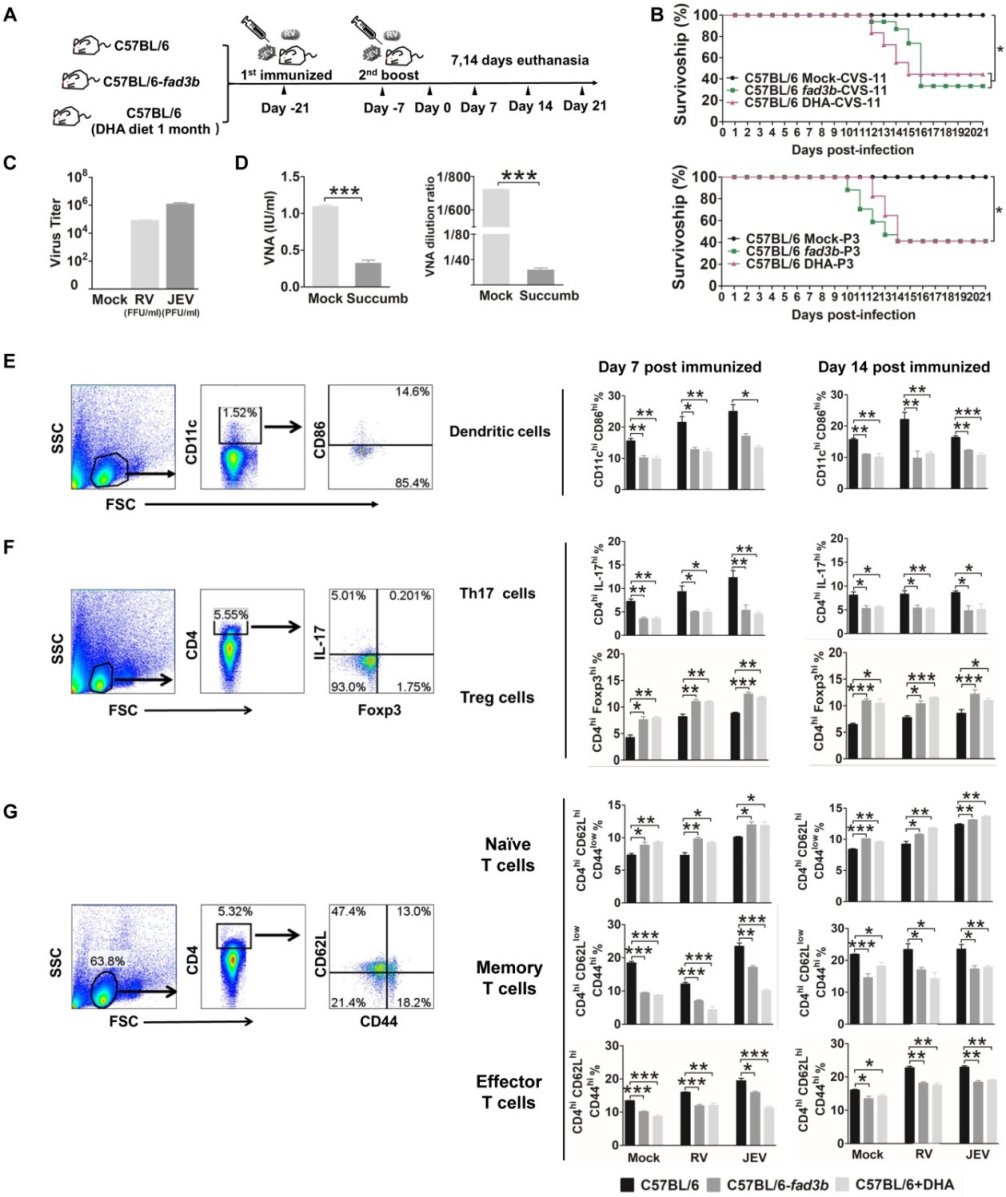
** Endogenous n-3 PUFAs and exogenous DHA inhibited DC and enhance the Tregs that reduce the protection of vaccination.** (A) Schematic illustration of the vaccination procedure for C57BL/6, C57BL/6-*fad3b*, and DHA-supplemented C57BL/6 mice. (B) Survivorship of mice infected with RV (top) and JEV (bottom). The statistical significance in the survival rates was analyzed by Kaplan-Meier plots (*n*=10 in each group, log-rank *P* < 0.05). (C) Virus titers of succumbed mice. (D) Average VNA of RV (left) and JEV (right) production of the surviving and succumbed mice. (E) DC were collected from the spleen at 7 and 14 days after immunization with RV or JEV and stained with fluorescently labeled antibodies against CD11c and CD86. (F) Flow-cytometric analysis of the proportion of CD4^+^IL-17A^high^ Th17 cells and CD4^+^Foxp3^high^ Treg cells. Splenocytes were stained with fluorescently labeled antibodies against CD4, IL-17 and Foxp3. (G) Flow-cytometric analysis of the proportion of CD4^+^CD44^low^ CD62^high^ naïve T cells, CD4^+^CD44^high^ CD62^low^ memory T cells, and CD4^+^CD44^high^ CD62^high^ effector T cells. Splenocytes were stained with fluorescently labeled antibodies against CD4, CD44, and CD62L.

We extended our observations by evaluating whether exogenous and endogenous DHA affect vaccination (JEV vaccine: SA-14-14-2 and RV vaccine: LBNSE) and immune cell differentiation in WT C57BL/6 mice supplemented with 500 mg/d dietary DHA or *fad3b* mice (Fig. 3A). After 21 days immunized with live JEV or RV, there were no neurological symptoms for the immunized mice. Seven days after the boost, all the mice were challenged with 50 LD50 of CVS-11 or 50 LD50 of P3 and observed for clinical signs and death for 21 days. As shown in Fig. 3B top, significantly fewer mice (40%) treated with DHA or *fad3b* mice (30%) survived the challenge of RV than mock-treated mice (100%) (*P*<0.05). As shown in Fig. 3B bottom, significantly fewer mice (40%) treated with DHA or *fad3b* mice (40%) survived the JEV challenge than mock-treated mice (100%) (*P*<0.05). In mice that succumbed to infection, RV virus titers reached 10^5^ FFU/ml, and JEV virus titers reached 10^6^ PFU/ml, and the VNA level of RV was less than 0.5 IU/ml (0.23 to 0.4 IU), and the VNA level of JEV was less than 1:20 (Fig. 3C and 3D). In the mock-treated mice immunized with RV or JEV, an average RV VNA production of 1.125 IU/ml and JEV VNA production of 1:720 was detected, and no RV virus was found in the brain of the survivors. These results demonstrate that exogenous or endogenous DHA supplementation failed to induce the production of VNA and protect mice against fatal challenge infections, while the mock-treated mice immunized with the vaccine strain of JEV and RV resulted in the induction of VNA and, subsequently, protection against challenge.

We next explored DC, T cell and B cell differentiation at seven days or 14 days post-booster immunization. The proportion of CD11c^+^ cells in the spleen of DHA supplemented mice and *fad3b* mice were similar to WT mice. Consistent with our *in vitro* findings, DHA supplementation *in vivo* significantly decreased the expression of CD86 on DC (Fig. 3E). Given the critical role of DC maturation for the efficient priming of T cells, we investigated whether DHA modulates the T cells phenotype. The proportion of CD4^+^Foxp3^+^ Tregs on day 7 and 14 hpi in the spleen was significantly enhanced after treatment with DHA or in *fad3b* mice compared with WT C57BL/6 mice. The increase of CD4^+^Foxp3^+^ Tregs was accompanied by a decrease in CD4^+^IL-17^+^ T cells (Fig. 3F). Moreover, DHA supplementation both significantly decreased the ratio of central memory T cells and effector T cells (Fig. 3G). However, DHA supplementation did not alter the proportion of CD138^+^ B cells (Fig. S3A, B). In agreement with our *in vitro* results, the excess exogenous and endogenous DHA supplementation keeps the DC in a resting state while enhancing the proportion of Tregs and reducing the protection conferred by the JEV or RV vaccine.

### Virus-driven MyD88 and NF-κB enhance FA metabolism and inhibit FFAR expression

**Figure 4:**
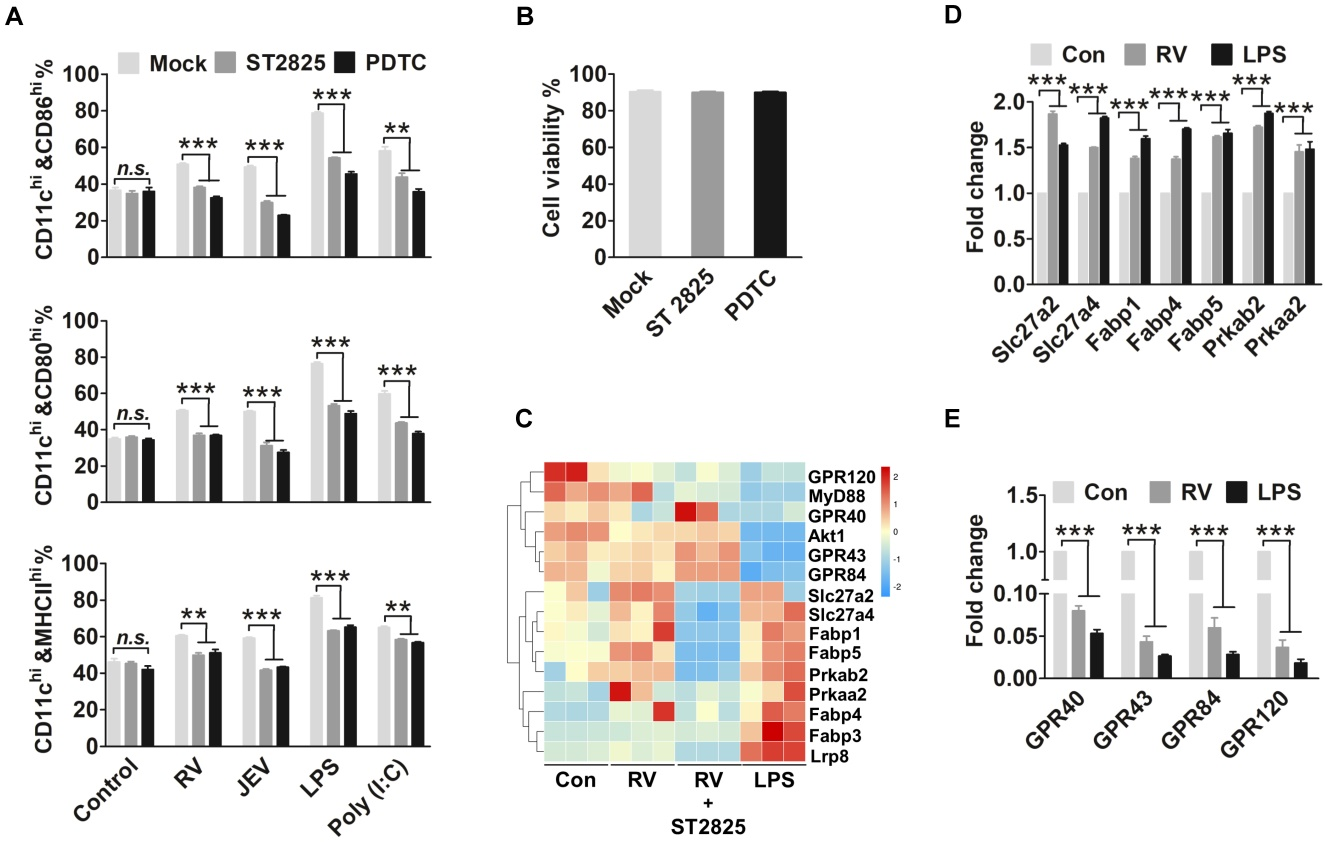
** MyD88 and NF-κB facilitate FA metabolism and inhibit FFAR expression.** (A) Flow-cytometric analysis of the proportion of CD86, CD80, and MHCII in CD11c-positive cells that are stimulated by RV, JEV, LPS, and Poly (I:C) for 24 h, and then incubated with ST2825 or PDTC for 12 hpi. (B) Analysis of DC viability after treatment with ST2825 and PDTC using trypan blue. (C) Changes in mRNA expression in DC stimulated with RV, RV and ST2825, and LPS. After 48 h of treatment, the miRNA expression level was measured by RNA deep sequencing and compared with those for the mock-treated control. The color scale is based on log_2_ changes in expression. (D) Quantitative RT-PCR (qRT-PCR) analyses of the fatty acid transporter protein expression levels in DC stimulated with RV and LPS. (E) Quantitative RT-PCR (qRT-PCR) analyses of the fatty acid receptor proteins.

Our results so far suggested that DHA is an essential cofactor that alters viruses and drives DC maturation. We hypothesized that the TLR and adaptor protein facilitate DHA metabolism. Next, to determine whether MyD88 and NF-κB are involved in the regulation of FA biosynthesis in DC, specific inhibitors were used to block these adaptor proteins. The immature bmDC were incubated with ST2825 or PDTC to inhibit the MyD88 or NF-κB 12 hpi. There was a significant 2-fold downregulation in CD86, CD80 and MHCІІ expression of ST2825 or PDTC treated in bmDC compared with mock-treated DC that were stimulated by RV, JEV, LPS and Poly (I:C) (Fig. 4A). Moreover, there was no change in cell viability between the ST2825 or PDTC treated, and untreated DC (Fig. 4B). The FA accumulation was blocked with ST2825 or PDTC treatment (Fig. S4A-D).

Our evidence suggests that FA accumulation is necessary for DC responses to viral infection. However, excess DHA supplementation effectively inhibits DC maturation. Thus, we performed RNA-seq analysis to search for the genes altered to reverse the inhibition of DHA and promote DC activation. The family of fatty acid transport proteins and fatty acid biosynthesis enzyme genes, such as *Slc27a2*, *Slc27a4*, *Fabp1*, *Fabp4*, *Fabp5*, *Prkaa2* and *Prkab2*, were increased in RV infected or LPS treated DC, confirming that FA accumulated during DC proliferation. However, the expression of free fatty acid receptors, such as GPR40, GPR43, GPR84, and GPR120, was markedly decreased (Fig. 4C). The RNA-seq data was confirmed with qRT-PCR (Fig. 4D and E). To verify that FA accumulation and FAs sensor associated genes were regulated by TLR and the adaptor protein in DC, the genes associated with FA metabolism and FA content were determined after treatment with ST2825 or PDTC. The genes associated with FA metabolism and FFARs that were treated with ST2825 were similar to those in medium-treated control cells. Also, there was no significant difference in FA content between TLR blocked cells and normal DC. These results suggest that virus-driven DC maturation not only promote FA metabolism but also reduce the expression of FFARs.

### DHA maintains DC in a resting-state via activation of GPR120

**Figure 5:**
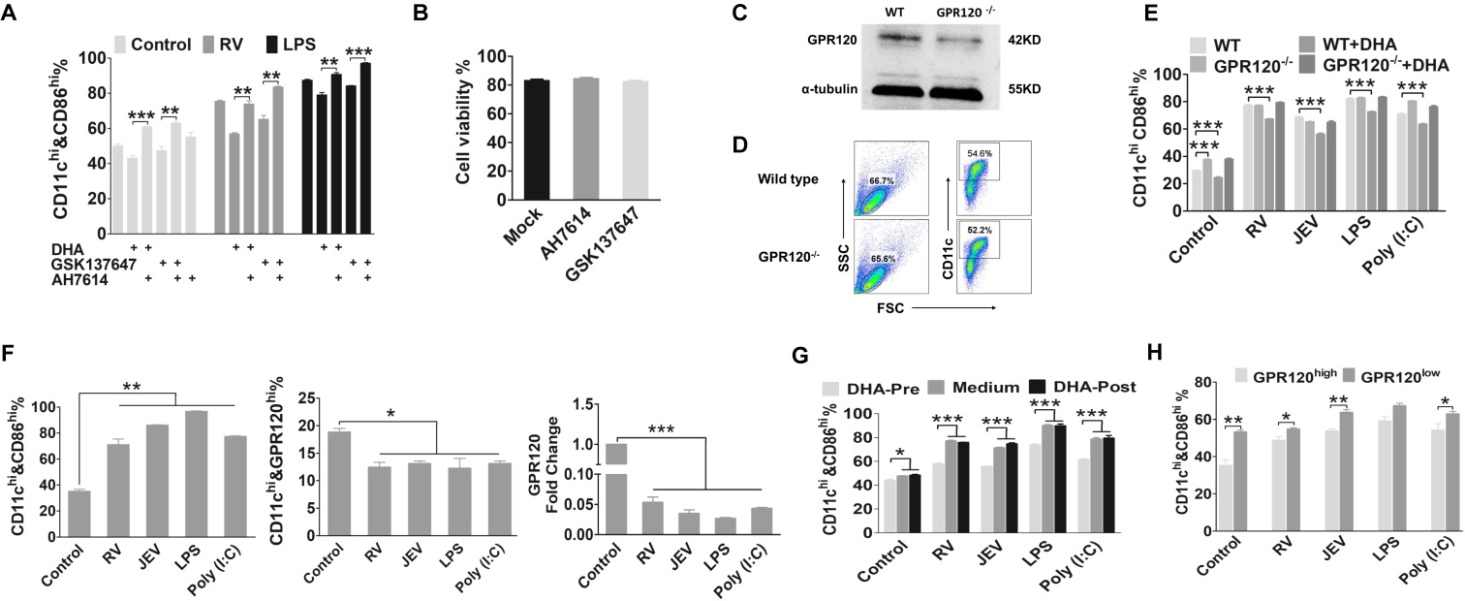
** DHA maintained DC in a resting-state through GPR120.** (A) Flow-cytometric analysis of the proportion of CD86 in CD11c-positive cells that blocked GPR120. DC were treated with DHA, GSK137647 (a GPR120 agonist), or AH7614 (a GPR120 inhibitor) for 6 h, and then stimulated by RV or LPS. After 24 h treatment, the DC were stained with fluorescently labeled antibodies against CD11c and CD86. (B) Analysis of DC viability after treated with AH7614 or GSK137647 using trypan blue. (C) Western blot analysis of the GPR120 expression on DC from wild-type and GPR120^-/-^ mice. Anti-GPR120 antibody was used to detect the expression of GPR120. (D) Flow-cytometric analysis of the proportion of the bone marrow derived cell and CD11c positive from wild-type and GPR120^-/-^ mice. (E) Flow-cytometric analysis of the proportion of the CD86 in CD11c-positive cells that isolated from GPR120^-/-^ mice. DC from wild-type C57BL/6 or GPR120^-/-^ mice incubated with DHA or mock medium for 6h, then DC were stimulated by RV, JEV, LPS, or Poly (I:C). After 24 h treatment, the DC were stained with fluorescently labeled antibodies against CD11c and CD86. (F) Flow-cytometric analysis and qRT-PCR analyses of the expression level of GPR120 on DC. DC with RV, JEV, LPS, and Poly (I:C) for 24 h and analyzed by flow cytometry staining with fluorescently labeled antibodies against CD86 and GPR120 (top). The RNA was isolated, and the expression level of GPR120 analyzed using qRT-PCR (bottom). (G) Flow-cytometric analysis of the proportion of CD86 in CD11c positive cells that treated with DHA before (pre) or after (post) stimulated by RV, JEV, LPS, and Poly (I:C). (H) Flow-cytometric analysis of the proportion of the CD86 in CD11c-positive cells that highly express GPR120, or show low expression of GPR120. The bmDC were stained with anti-GPR120 and sorted into the highly expressing GPR120 subtype or low expressing GPR120 subtype. After sorting, the two DC subtypes were stimulated with RV, JEV, LPS, and Poly (I:C) for 24 h, and the CD86 expression analyzed by flow cytometry.

Previous studies have reported that GPR120 is a specific receptor of long-chain FAs, such as DHA, which decreases inflammation responses in macrophages (12,29). To specifically address the role GPR120 in DC, we assessed the effect of a GPR120 agonist (GSK137647) and antagonist (AH7614) on the maturation markers in DC. As shown in Fig. 5A, the proportion of CD11c^+^ CD86^+^ cells was significantly reduced following pre-treatment with DHA and GSK137647 in both viral infected and mock-infected DC. In contrast, DHA did not affect the expression of the maturation marker in AH7614-treated DC. Hence GPR120 is necessary for DHA to alter the maturation state of DC. Moreover, there was no change in cell viability between the GSK137647 and AH7614 treated, and untreated DC (Fig. 5B). To further confirm these results and to verify the GPR120 specificity, we constructed a GPR120 knockout mice and the GP120 expression level in DC was lower than WT mice (Figure 5C). Furthermore, the amount of bone marrow derived cells and CD11c+ cells that generated from GPR120 knockout mice bone marrow was similar to the WT mice (Figure 5D). Similarly, DC generated from GPR120^-/-^ mouse bone marrow was infected with JEV and RV, in which the DHA treatment had no significant effect on the expression of CD86, CD80, or MHCII (Fig. 5E). However, the proportion of maturation markers on the resting state of DC generated from GPR120^-/-^ mouse bone marrow was significantly higher than the DC generated from WT mice (Fig. 5E). Hence, these results support that DHA maintains DC in a resting state primarily through GPR120.

Our results implied that virus-driven MyD88 and NF-κB induced the reduction of GPR120. We hypothesized that a decrease in GPR120 expression blocks the DHA-induced inhibition on DC that in turn, promote their maturation. Because of the GPR120 expression during maturation, DC were significantly fewer in number than the immature DC (Fig. 5F). Hence, we evaluated whether DC maturation could be altered by DHA. After 24 hours of virus infection, DHA supplementation could not decrease the expression of maturation markers on DC. This finding implied that a mature DC presents less GPR120, which is not sensitive to stimulation by DHA (Fig. 5G). In addition, the immature DC generated from WT C57BL/6 mice could be separated into two subsets that showed either high or low GPR120 expression. After sorting and DHA treatment, the maturation marker on DC was measured, and the maturation markers on high GPR120 expression DC were all significantly decreased (Fig. 5H). Strikingly, DHA did not affect low GPR120 expression DC. In contrast, the high GPR120 expression DC were more sensitive to the virus, LPS, and Poly (I:C). Furthermore, there are no significant differences in maturation markers on the high GPR120 expression DC after viral infection compared with mock infection control DC. This finding implies that reducing the expression of GRR120 facilitates the maturation of DC and GPR120^low^ DC exhibited higher sensitivity to viral infection than GPR120^high^ DC.

### The deletion of GPR120 enhances the spontaneous maturation of DC and blocks antigen presentation

**Figure 6:**
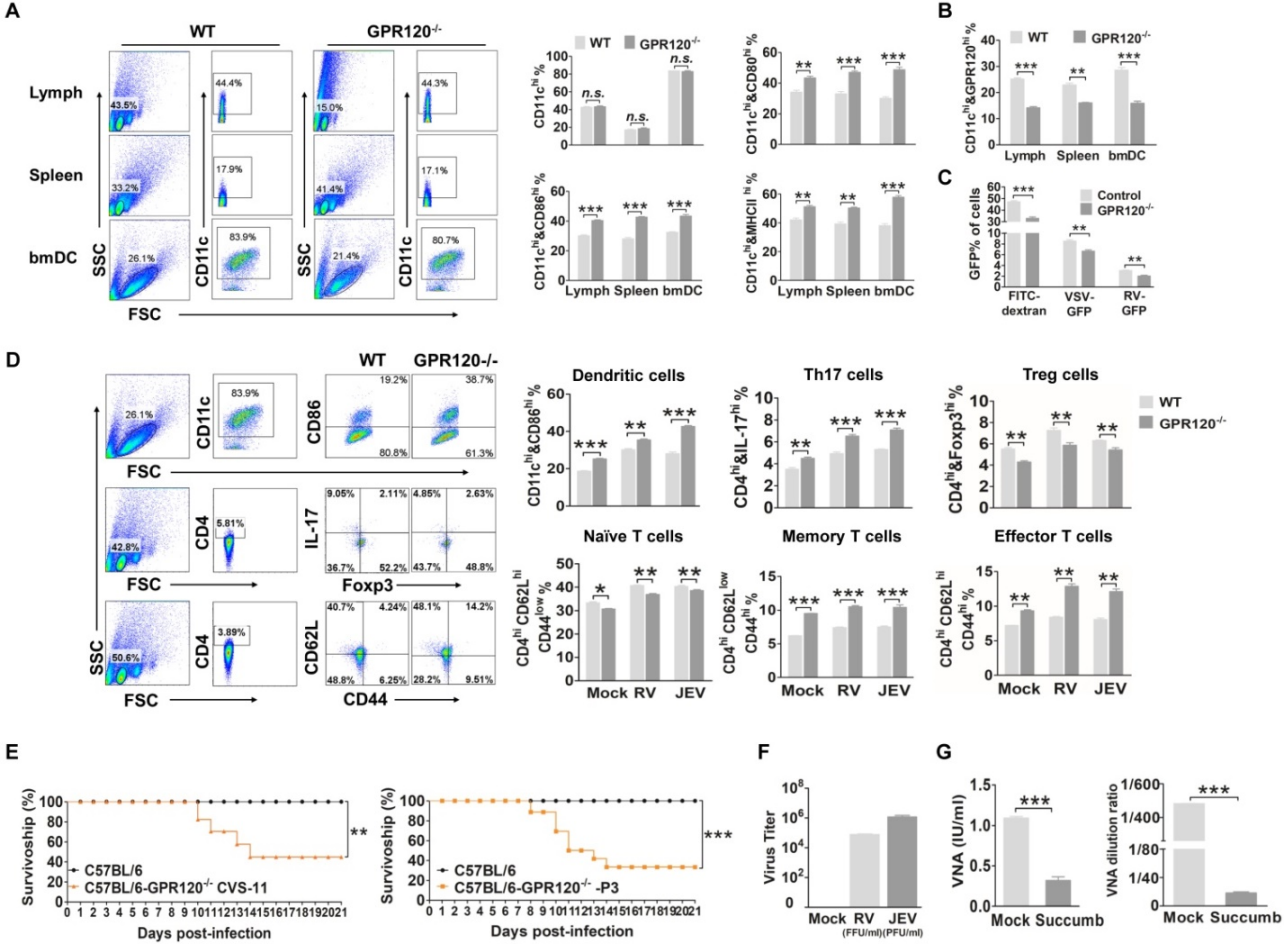
** GPR120 is required for viral-induced DC maturation and skews Treg/Th17 balance.** (A) Flow-cytometric analysis of DC isolated from GPR120^-/-^ mice. DC were collected from the lymph, spleen or bmDC of GPR120^-/-^ C57BL/6 mice or WT C57BL/6 mice, and stained with fluorescently labeled antibodies against CD11c, CD86, CD80, and MHCII. (B) The expression of GPR120 on bmDC, splenocytes and leukocytes isolated from GPR120^-/-^ and WT mice analyzed by flow cytometry. (C) Flow-cytometric analysis of macropinocytosis using FITC-dextran, VSV-GFP, or RV-GFP in DC isolated from GPR120^-/-^ C57BL/6 mice or WT C57BL/6 mice. (D) Flow-cytometric analysis of proportion of the CD4^+^IL-17A^high^ Th17 cells, CD4^+^Foxp3^high^ Treg cells, CD4^+^CD44^low^ CD62^high^ naïve T cells, CD4^+^CD44^high^ CD62^low^ memory T cells, and CD4^+^CD44^high^ CD62^high^ effector T cells. Splenocytes were stained with fluorescently labeled antibodies against CD4, IL-17, Foxp3, CD44 and CD62L. (E) Survivorship of C57BL/6 and GPR120^-/-^ mice infected with RV (left) and JEV (right). (F) Virus titers of succumbed mice. (G) Average VNA of RV (left) and JEV (right) production in the surviving and succumbed mice. The statistical significances of survival rates were analyzed using Kaplan-Meier plots (*n*=10 in each group, log-rank *P* < 0.05). For all graphs, results are shown as the mean ± SEM. **p <0.01 and ***p <0.001 based on a Student's* t-*test.

Given the importance of FAs special DHA is not only a critical cellular constituent but also a cellular signaling molecule, and lack of FAs was hardly to generate DC, we used GPR120^-/-^ mice to determine whether DHA deficiency-associated signaling disordered DC maturation processing. We observed that C57BL/6 GPR120^-/-^ mice have no overt developmental, behavioral or physiological defects, and display a similar classification of leukocytes in the spleen and lymph when compared with WT C57BL/6 mice (Fig. 6A). Consistent with our *in vitro* results, the CD11c^+^CD86^+^, CD11c^+^CD80^+^, and CD11c^+^MHCII^+^ cells in the lymph, spleen, and bmDC of GPR120^-/-^ mice was significantly higher than the WT C57BL/6 mice, respectively. Also shown in Fig. 6B, GPR120 expression on the surfaces of CD11c^+^ cells in the lymph, spleen, and bmDC in GPR120^-/-^ mice was 11%, 7%, and 14% lower than in WT C57BL/6 mice, respectively. Furthermore, the capacity for macropinocytosis of GPR120^-/-^ mouse derived DC was determined by FITC-dextran, VSV-GFP, or RV-GFP. GPR120^-/-^ mouse derived DC presented a significantly lower uptake of FITC-dextran and virus than normal DC (Fig. 6C).

Our data suggest that decreased GPR120 expression induced spontaneous maturation of DC, which would result in a blunted activated response to the vaccine. Thus, the GPR120 deficient mice were used to test whether DHA deficiency affects vaccine induction of the anti-viral immune response. The GPR120^-/-^ mice and WT C57BL/6 mice were vaccinated twice with rabies or Japanese encephalitis (JE) vaccine (21 days before infection and seven days before infection, respectively). Seven days after the boost, all the mice were challenged with 50 LD50 of CVS-11 or 50 LD50 of P3 and observed for clinical signs and death for 21 days. Consistent with our *in vitro* findings, the deletion of GRP120 *in vivo* significantly enhances the expression of CD86 on DC in the spleen (Fig. 6D). Next, we investigated whether deletion of GRP120 modulates the T cell phenotype. The proportion of CD4^+^IL-17^+^ T cells seven days post-boost in the spleen was significantly enhanced in the GPR120^-/-^ mice compared with the WT C57BL/6 mice. Conversely, CD4^+^Foxp3^+^ Tregs of the GPR120^-/-^ mice were significantly decreased. Moreover, the deletion of GRP120 both significantly increases the ratio of central memory T cells and effector T cells (Fig. 6D). The deletion of GRP120 did not alter the percentage of CD138^+^ B cells (Fig. S5).

One day after the boost, all mice were challenged with 50 LD50 of CVS-11 or 50 LD50 of P3 and observed for clinical signs and death for 21 days. As shown in Fig. 6E, C57BL/6 GPR120^-/-^ mice (40%) survived the challenge, and C57BL/6 WT mice survived the RV challenge (100%) (*P*<0.05). Furthermore, C57BL/6 GPR120^-/-^ mice (30%) survived the challenge, and C57BL/6 WT mice survived the JEV challenge (100%) (*P*<0.05). In mice that succumbed to rabies, virus titers reached 10^5^ FFU/ml, and the VNA level was less than 0.5 IU/ml (Fig. 6F-G). In mice that succumbed to JE, virus titers reached 10^6^ FFU/ml, and the VNA level was less than 1:20. In the mice that survived RV or JEV challenges, an average RV VNA production of 1.25 IU/ml and JEV VNA production of 1:480 was detected, and no RV or JEV was found in the brains of the survivors. These results demonstrate that GPR120 deficiency reduces the production of VNA and protects mice against challenge infections, while the mock-treated mice immunized with the vaccine strain of JEV and RV resulted in the induction of VNA and, subsequently, protection against challenge. Collectively, these findings indicate that GPR120 deficiency induces DC spontaneous maturation and leads to vaccine failure.

## Discussion

The metabolic factor that controls the immunosuppressive subtype of DC, and its impact on T cell differentiation is still obscure. In this study, our results demonstrate that GPR120 plays a crucial role in regulating the DC phenotype. FA metabolism is necessary for DC generation, and DHA suppressed DC maturation through GPR120 and skewed the Treg-Th17 balance *in vivo* in favor of Tregs. The maturation of DC reduce GPR120 expression and decrease DHA-induced inhibition of DC. However, the absence of GPR120 markedly promoted the proportion of spontaneously maturing DC and reduced the antigen uptake of DC. Our findings reveal that both DHA supplementation and the deletion of GPR120 were not only associated with decreased immune effects of the vaccine but also enhanced viral infection.

DC activation with proinflammatory stimuli elevates surface maturation markers which could be blocked by DHA, highlighting a role for DHA in regulating DC maturation 18, 43. Our data are in agreement with those that show DHA supplementation inhibits maturation of DC *in vivo* and *in vitro*, and decreases the protection of JEV and RV immunization. It is known that immunosuppression decreases the protection of vaccines and renders patients as high risk for infection, and live attenuated vaccine might lead to infection in immunosuppressed patients 9, 44. Although our findings revealed that DHA-induced immunosuppression did not cause live attenuated JEV or RV infection, the survivorship of DHA-treated mice after immunization with the vaccine was markedly lower than in mock-treated mice. Furthermore, we demonstrate that DHA suppresses IFN secretion by DC, which could skew the Treg-Th17 balance. As expected, the proportion of Tregs was significantly improved following DHA supplementation and the expression level of VNA was markedly restricted, suggesting that DHA could alter T cell differentiation to block the anti-viral immune response. Also, Tregs facilitate viral infection by restricting antiviral cytokine production and inhibitory effects on cell trafficking of protective T cells to infected sites 45, 46. This result also agreed with previous work showing that DHA supplementation promotes pathogen infection 20, 23-26. Moreover, our findings reveal that DHA suppresses anti-viral immune responses by modulating DC maturation and T cell differentiation.

In previous reports, there is a near equal number of studies published that report an adverse effect of n-3 PUFA on host infectious disease resistance as those that show a beneficial effect 25. Several studies suggested that the DHA supplementary dose may play a critical role in promoting or inhibiting the immune response 47. The approximate DHA concentration at baseline in the blood was 33 μg/ml 48. We demonstrated that an added extra 33 μg/ml of DHA significantly inhibited DC maturation, and DC differentiation was blocked under the free FA conditions. Similarly, blockade of FA synthesis decreased DC expression of maturation markers has been reported 49. In particular, cellular metabolism has been identified as an essential regulator of DC development and antigen presentation 50, 51. To avoid the effects of DHA deficiency on the synthesis of cellular components, we used GPR120^-/-^ mice to determine whether DHA deficiency associated signaling disordered DC maturation processing. The maturation markers on GPR120-deficient DC were markedly higher than normal DC, and antigen uptake was lower than normal DC. It is further suggested that DHA is not only an essential cellular component but is also a critical signal to regulate DC differentiation. In addition, based on the American background diet, several expert groups have established recommendations that typically range from 250 mg/d to 500 mg/d EPA+DHA for general health and the mice in our study were treated with a similar amount of DHA 52, 53. In agreement with *in vitro* results, DHA inhibits DC maturation and promotes the proportion of Tregs *in vivo*. In particular, our data reveal that GPR120 deficiency promotes Th17 cell differentiation and attenuates Tregs without stimuli, which might block antigen processing and reduce VNA secretion. These results suggested that a proper dose of DHA promotes stimulation of the anti-viral immune response.

Several genes have been identified as promoting induction of spontaneous autoimmunity in DC upon their deletion 34, 35, 54. Although these genes are generally negative regulators of immune activation and their deletion in other cell types also induce spontaneous inflammation and autoimmunity. Especially for our study, the deletion of GPR120 not only caused the spontaneous maturation of DC but also induces Th17 proliferation and enhances the proportion of memory T cells and effector T cells. Previous studies reported that DC facilitates peripheral T cells tolerance to antigens and induces T cell-mediated autoimmunity 55, 56. Thus, the spontaneous maturation of DC powerfully induces a broad range of autoimmune responses.

In conclusion, we identified GPR120 as an inhibitor of DC maturation. DHA induces a tolerogenic DC phenotype and enhances the Tregs population *in vivo* through GPR120. We found that upon RNA viral infection in DC, the content of FAs, including DHA, was elevated while the expression of GPR120 was significantly decreased, and decreased GPR120 was associated with the maturation of DC. However, the deletion of GPR120 blocks the inhibition of DHA that promotes the spontaneous maturation of DC and decreases the proportion of Tregs. In particular, excessive activation of GPR120 by DHA or deletion of GPR120 effectively reduces the production of VNA and the protection resulting from vaccination. Whether DHA and GPR120 directly influence T cell differentiation still requires exploration. Nonetheless, our findings suggest that a proper supplement of DHA can favorably promote immune cell function. Thus, manipulating physiological DHA homeostasis and GPR120 agonists or antagonists could serve as an attractive therapeutic strategy for treating autoimmune diseases and improving vaccine effectiveness.

## Materials and Methods

### Ethics statement

Experimental infectious studies were performed in strict accordance with the Guide for the Care and Use of Laboratory Animal Monitoring Committee of Hubei Province, China, and the Scientific Ethics Committee approved the protocol of Huazhong Agricultural University (protocol No. Hzaumo-2015-018). All efforts were made to minimize the suffering of the animals.

### Cells, virus and mice

Mouse neuroblastoma (NA) cells were maintained in RPMI 1640 medium (Gibco, USA) supplemented with 10% fetal bovine serum (FBS) (Gibco). Baby Hamster Kidney fibroblasts (BHK-21) cells and BSR cells (a cloned cell line derived from BHK-21 cells) were maintained in Dulbecco's modified Eagle's medium (Gibco) containing 10% FBS. Bone marrow-derived dendritic cells (bmDC) were generated as previously described 57. Briefly, bone marrow was removed from tibias and femur bones of C57BL/6 mice. Following lysis of red blood cells with ACK lysis buffer, progenitor cells were plated in DC media [RPMI 1640 medium with 10% FBS, 20 ng/ml granulocyte-macrophage colony- stimulating factor (GM-CSF; PeproTech, USA)] in 100 mm Non-Treated Petri Dish at 10^6^ cells/ml. Half of the medium was discarded and replaced with fresh DC medium every other day. On day 7, loosely adherent cells were collected for analysis. DC were washed and variously stimulated with the virus or other stimulants. Flow cytometric analysis of purified DC displayed low levels of CD86, CD80 and MHC II expression which are characteristics of immature DC. Vaccine strain LBNSE of rabies virus was generated as described previously 58. Challenge virus standard CVS-11 strain of rabies virus was bred in the sucking mice brain as previously 59. JEV vaccine strain SA-14-14-2 was propagated in BHK-21cells and JEV wild type strain P3 was propagated in brains of suckling mice as previously 60. VSV labeled with a green fluorescent protein (GFP) (a gift from Ling Zhao, Huazhong Agriculture University, Wuhan, China) was propagated in NA cells. C57BL/6 and CD1 mice were purchased from Vital River Laboratories (China). The *fad3b* mice were maintained in our lab and described as previously 61. All mice were bred under specific pathogen-free conditions.

### Construction of GPR120^-/-^ mice

The GPR120^-/-^ mice were created in a C57BL/6 background and maintained in this background, which contains a 23bp deletion in the exon 1. The GPR120-sgRNA (5'-CCAAGTCAATCGCACCCACTTCC-3') and Cas9 mRNA were produced by *in vitro* transcription (IVT) using a machine SP6 Ultra Kit (Thermo, USA) according to the manufacturer's instructions. Seven-week old female C57BL/6 mice were super-ovulated via intraperitoneal injection of Pregnant Mare Serum Gonadotropin (PMSG, Sansheng, China) at 5 p.m. followed by Human Chorionic Gonadotropin (hCG, Sansheng) 48 h later and then mated with 12-week old male C57BL/6 mice immediately. Zygotes were collected from the oviducts 24 h after hCG injection, and Cas9 mRNA (50 ng/µl) and sgRNA (50 ng/µl) were microinjected into the cytoplasm of zygotes. Survived zygotes were cultured at 37℃ in 5%CO_2_ for two h and were transferred into the oviductal ampullas of the pseudopregnant CD1 mice. Genomic DNA from the tails of mice after weaning was subjected to PCR analysis by using Premix ExTaq with primer (GPR120-PCR-S: 5'-CAGCTCCTACGCCCAGAG-3', GPR120-PCR-A: 5'-CGCCGAGTAACCCCATAT-3'). The PCR products were sequenced and genotyped. To obtain pure unique homozygous KO mice, the F1 generation GPR120^+/-^ mice were crossed with wild-type C57BL/6 mice to generate a F2 generation. The F2 heterozygous GPR120^+/-^ mice (male and female) were intercrossed to breed a F3 generation. F3 newborns with homozygous mutations were confirmed by sequencing analysis (GPR120^-/-^).

### Virus titration

Virus titration of RV was performed with FITC-labeled anti-rabies virus N protein antibodies (FujiRebio Diagnostics Inc) in RV-infected NA cells as described previously 62. NA cells per well were cultured in a 96-well plate were inoculated with serial 10-fold dilutions of the RV and then incubated at 34°C for two days. Na cells then were fixed with 80% ice-cold acetone for 30 min, then washed twice with PBS and stained with FITC-conjugated anti-RV N antibodies (FujiRebio Diagnostic Inc). Antigen-positive foci were counted under a fluorescence microscope (Nikon, Japan), and virus titer was calculated as the number of focus-forming units per milliliter (FFU/ml). All titrations were carried out in quadruplicate.

### Viral plaque assay

JEV was titrated on BHK-21 cells line by viral plaque formation assay as previously 63. BHK-21 cells were cultured in 12-well for 12 h and then inoculated with 10-fold dilutions of JEVs. After 2 h of incubation, cells were washed with PBS and then cultured in DMEM with 1% FBS and 4% sodium carboxymethyl cellulose (Sigma, USA). After five days incubation at 37°C, the cells were fixed with 10% formaldehyde overnight, followed by staining with crystal violet for 2 h. Visible plaques were counted, and the virus titer was calculated as the number of plaque-forming units per milliliter (PFU/ml). All data were calculated as the means of triplicate.

### IFN sensitivity assay

DC supernatants were assessed for type I IFN induced by JEV, RV, LPS, and Poly (I:C) with an indirect method as described before 64. Briefly, after 24 h treatments, the DC supernatant was collected and deactivated with a 254 nm UV light source for 30 min. A reporter cell line (NA cells) was treated with UV-inactivated viral supernatants (diluted 1:10) and then infected with VSV-GFP at a MOI of 1 for 6 h. VSV replication was examined by fluorescence microscopy, and the type I IFN production was inversely correlated with GFP expression.

### Viral infection in DC

DC were plated in 12-well plates at 5×10^5^ cells/well and then incubated with DHA, GSK137647, AH7614 or Mock treated with RPMI 1640. After incubation for 6 h, DC were inoculated with JEV (SA), RV (LBNSE) or mock treated with RPMI 1640, and cultured for another 24 h. For the analysis of DC activation, cells were stained with specific fluorescent-conjugate antibodies against CD86, CD80, and MHCII to analyze by BD Accuri™ C6 flow cytometry (BD, USA), and the supernatant was analyzed by IFN sensitivity assay.

### FACS analysis

DC differential markers and fluorescent-labeled cells were measured by flow cytometry, which was carried out as described previously 65, with some modifications. Cells were suspended with PBS contained 1% FBS and 2 μM EDTA solutions and blocked with rat anti-mouse CD16/CD32 (Fc block) (BD) at 1:100 dilution. DC were stained with isotype control, anti-CD11c-APC, anti-CD80-PE, anti-CD86- FITC, and anti-MHCII-PerCP-Cy5.5 antibodies for 1 h at 37℃ with gentle shaking every 10 min. All antibodies were purchased from BD. Mouse Th17/Tregs phenotyping kit and mouse Naïve/Memory T cell panel was used to differentiate T cell subtypes. For Foxp3 staining, cells were treated with Mouse Foxp3 Permeabilization Buffer (BD) and then stained with Th17/Tregs phenotyping cocktail (BD). For BODIPY staining, cells were washed with PBS to remove the medium. BODIPY 493/503 labeled lipid droplets in DC (2 μM, Thermo) were incubated at 37℃ for 15 min in the dark. FACS data collection were performed by BD C6 flow cytometer with BD Accuri C6 Software (BD), and data were analyzed with flow Jo software (Tree Star, San Carlos, CA).

### Phagocytosis assay

The phagocytosis ability of DC was measured via uptake of FITC-dextran, RV-GFP, and VSV-GFP. 2×10^5^ DC were resuspended in 500 µl DC culture medium, supplemented with 10 μg/ml FITC-dextran, 10 MOI of RV-GFP or VSV-GFP for 1 h at 37°C. After incubation, the cells were washed three times and resuspended with ice-cold PBS with 2 μM EDTA, and then analyzed by flow cytometry.

### Cells sorting by FACS

Isolation of CD11c^+^ GPR120^+^ DC with flow cytometry sorting. GPR120 primary antibody (Abcam, UK) was added to stain the GPR120 at RT for 1 h, followed with twice PBS washing and stained with anti-mouse IgG-FITC and anti-mCD11c-APC antibodies to the mice single-cell suspension, incubated it at RT for 1 h. After the incubation, the cells were washed with PBS for three times and adjusted the cell concentration to 1×10^7^/ml. The CD11c^+^ GPR120^+^ DC were sorted with FACS Aria II SROP (BD), and then CD11c^+^ GPR120^+^ DC and CD11c^+^ GPR120^-^ DC were separately in the two flow tubes to perform other experiments.

### Fatty acids treatment

Fatty acids (FAs) were purchased from Cayman and diluted in ethanol for all FAs [Palmitic acid (PA, C16:0), stearic acid(C18:0), palmitoleic acid (C16:1), oleic acid (OA, C18:1n-9), linoleic acid (LA, C18:2n6c), cis-8,11,14-eicosatrienoic acid (C20:3n6), cis-11,14,17- eicosatrienoic acid (C20:3n3), erucic acid (C22:1n9), eicosapentaenoic acid (EPA, C20:5n3), nervonic acid (C24:1n9), docosahexenoic acid (DHA, C22:6n3) and arachidonic acid (AA, C20:4n-6)] . The FAs-free medium was contained RPMI-1640 basic medium, 1×ITS medium supplement (Sigma) and 20 ng/ml GM-CSF without FAs supplement. Before addition to the culture medium, all FAs were conjugated to bovine serum albumin (BSA, fatty acid-free, Sigma) at the indicated concentration with FAs-free medium containing 1% BSA. The fatty acids were incubated with 1% BSA for 2 h before adding to DC. The concentration of saturated, monounsaturated, and polyunsaturated fatty acids in ITS medium was 20 µM. Half of the medium was discarded and replaced with fresh DC medium every other day. On day 6, DC were infected with the virus for 24 h and then analyzed by FACS.

### ST2825 and PDTC treatment

ST2825 and PDTC were purchased from Apexbio (Houston, TX, USA) and dissolved in dimethylsulfoxide (DMSO). DC were plated in 60 mm dishes at 3×10^6^ cells/dishes and then incubated with 30 µM ST2825 or 100 µM PDTC. After incubation for 6 h, DC were incubated with JEV (SA-14-14-2), RV (LBNSE), LPS, Poly (I:C) or blank medium. After 24 h incubation, the DC were analyzed by FACS.

### BODIPY and immunofluorescent staining for microscopy

The coverslips were treated with collagen and washed with PBS, and then incubated with the virus and other stimulants for 24 h. The cells were incubated in the staining solution (2 μM BODIPY staining solution in PBS) at 37℃ in the dark. After 15 min staining, the cells were washed twice with PBS and fixed with 4% paraformaldehyde for 30 min at room temperature. Coverslips were then washed twice with PBS and stained with DAPI. The images were analyzed with Confocal Microscope (A1R, Nikon, Japan).

### Gas chromatography-mass spectrometer (GC-MS) analysis

Cells were collected and washed with PBS. Then, cells were treated with 1 ml methanol solution with 2.5% H_2_SO_4_ at 80°C. After 90 min, cells were treated with 1.5 ml 0.9% NaCl and vortexed at RT for 5 min. Next, cells were treated with 0.4 ml saturated KOH methanol solution and centrifuged for 10 min at 2000 rpm/min. The fatty acid methyl esters (FAMEs) were collected into a clean vial. The FAME analysis was performed on Gas Chromatography-Mass Spectrometer (GC-MS) (Shimadzu GCMS-QP2010 Ultra, Japan) equipped with an autosampler injector (AOC-20i) and an electron-impact ionization mass spectroscopy.

### RNA sequencing

Total RNA was extracted from culture DC using the Total RNA Extractor Kit (B511311, Sangon, China) according to the protocols. A total of 2 μg RNA from each sample was used for library preparation according to the manufacturer's instructions for the VAHTSTM mRNA-seq V2 Library Prep Kit for Illumina®. PCR products were purified (AMPure XP system) and library quality was assessed on the Agilent Bioanalyzer 2100 system. Paired-end sequencing of the library was performed on the HiSeq XTen sequencers (Illumina, San Diego, CA).

### Quantitative Real-time PCR assay

The total RNAs were isolated from cultured cells using TRIzol reagent (Thermo) according to the manufacturer's instructions and were treated with DNase I (Thermo). cDNA was prepared from total RNAs using PrimeScript^TM^ RT reagent Kit with gDNA Eraser (Takara, Japan). qRT-PCR was performed using SYBR Premix ExTaq (Takara) and analyzed on an ABI PRISM 7500 real-time cycler (Thermo). Primers for PCR were listed in Table S1. Amplification of endogenous *Gapdh* was used as an internal control.

### Statistical analysis

All data were analyzed with GraphPad Prism software (GraphPad Software, Inc., CA). An unpaired two-tailed *t*-test was used to determine the statistical significance of the infection ratio and virus titers in transgenic cell lines. Experiments to determine the percentage of survival, Kaplan-Meier survival curves were analyzed by the log-rank test. For all tests, the following notations were used to indicate significant differences between groups: *P < 0.05; **P < 0.01; ***P < 0.001.

## Supplementary Material

Supplementary figures and tables.

## Abbreviations

DHA: docosahexenoic acid
GPR120: G protein-coupled receptor 120
DC: dendritic cell
APCs: antigen present cells
PRRs: pattern recognition receptors
FA: fatty acid
n-3 PUFAs: n-3 polyunsaturated fatty acids
BM: bone marrow
RV: rabies virus
JEV: Japanese encephalitis virus
LPS: lipopolysaccharide
IFN: interferon
Poly (I:C): polyinosinic acid-polycytidylic acid
VNA: virus neutralizing antibody
WT: wild-type
FBS: fetal bovine serum
BHK-21: Baby Hamster Kidney fibroblasts cells
BSR: a cloned cell line derived from BHK-21 cells
DMEM: Dulbecco's modified Eagle's medium
FAMEs: fatty acid methyl esters
DMSO: dimethylsulfoxide
