# Supplementary material for: DHA Sensor GPR120 in Host Defense Exhibits the Dual Characteristics of Regulating Dendritic Cell Function and Skewing the Balance of Th17/Tregs
Source: Int J Biol Sci. 2020 Jan 1;16(3):374–87. doi: 10.7150/ijbs.39551 (PMC6990895; doi:10.7150/ijbs.39551)
Supplement: Supplementary file 1 — Supplementary figures and tables. [file ijbsv16p0374s1.pdf]

Supplementary Materials

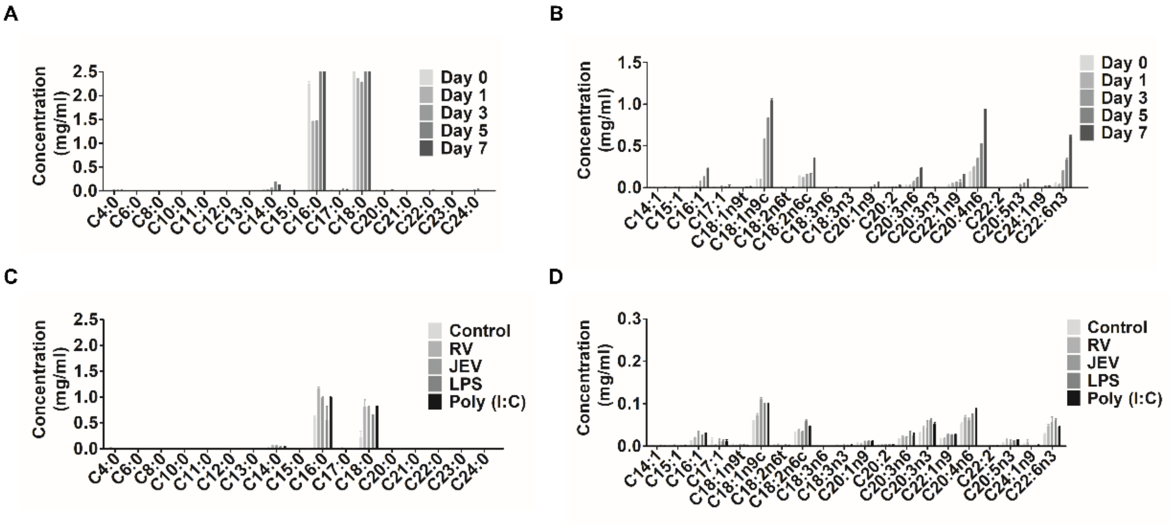

Figure S1. Changes of fatty acids in DC during generation and activation analyzed by GC-MS. (A-B) The saturated fatty acids and unsaturated fatty acids changes of DC at day 0, 1, 3, 5 and 7. (C-D) The saturated fatty acids and unsaturated fatty acids changes in DC after stimulated by RV, JEV, Poly (I:C), and LPS for 24 h.

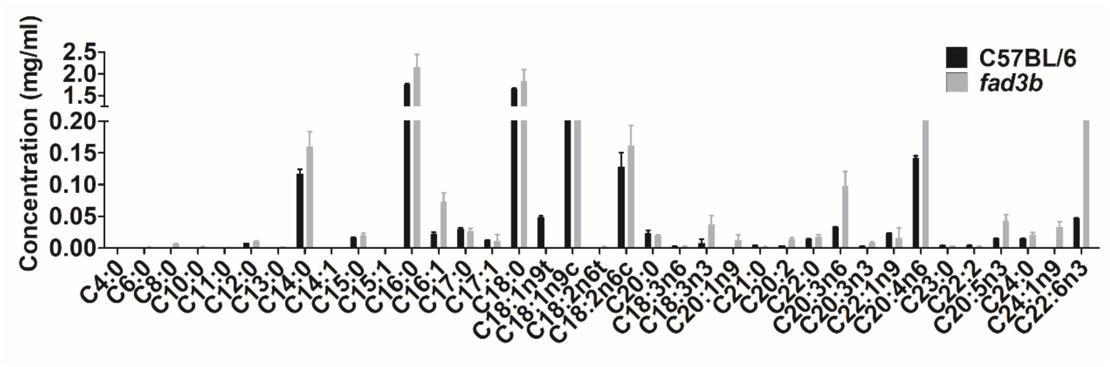

Figure S2. Changes of 37 types of fatty acids in DC. DC derived from C57BL/6 and *fad3b* mice after cultured 7 days and extract FAs analyzed by GC-MS.

A B

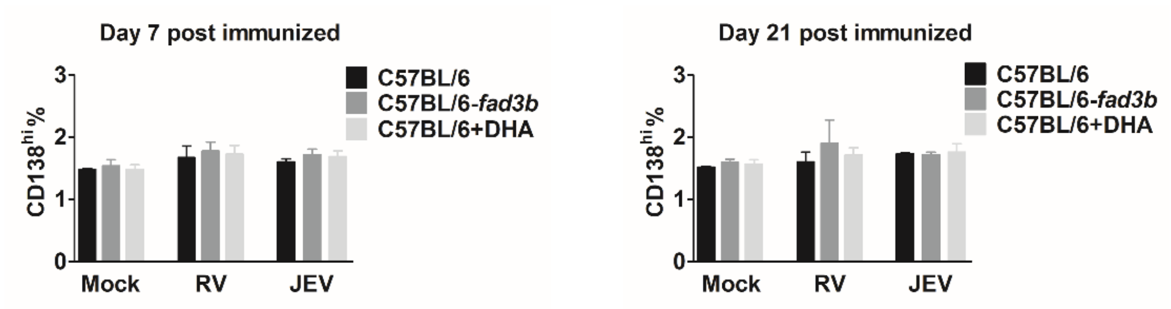

Figure S3. Flow-cytometric analysis of the proportion of the CD138 in CD11c positive cells after treated with RV, JEV. DC were collected from the spleen at 7 and 21 days after immunized by RV and JEV and stained with fluorescently labeled antibodies against CD11c and CD138.

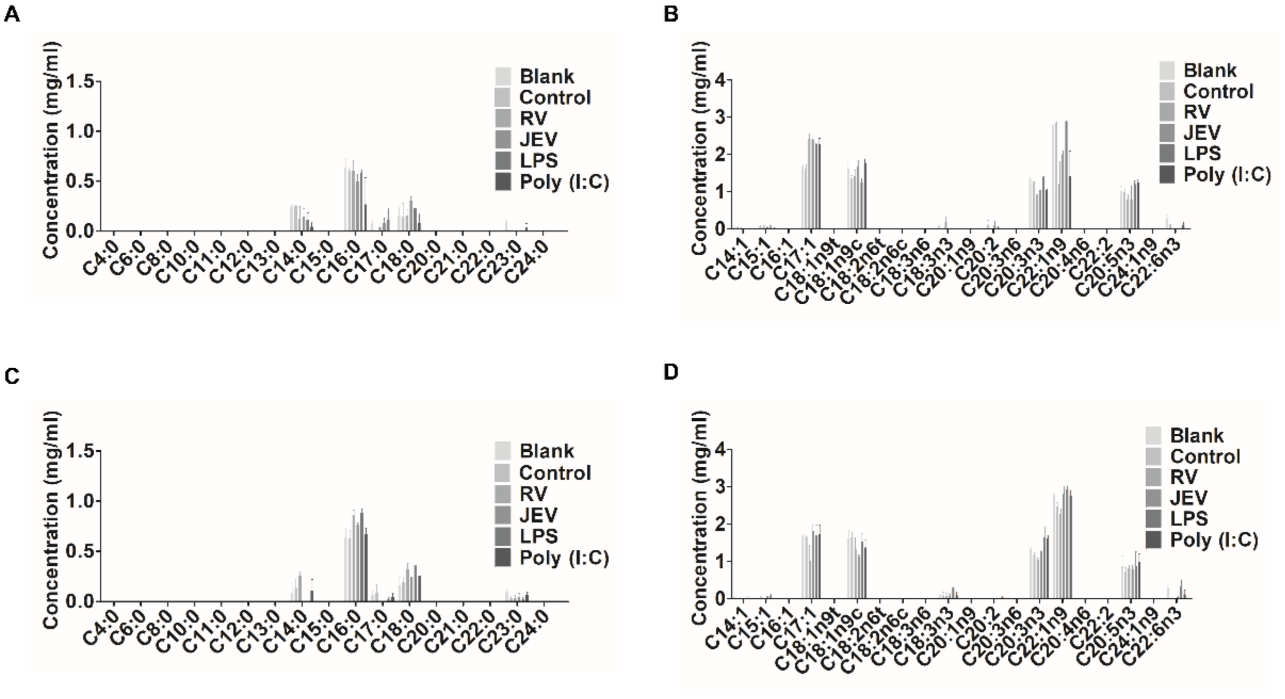

Figure S4. Changes of fatty acids in DC after treated by ST2825 and PDTC which analyzed by GC-MS. (A-B) The saturated fatty acids and unsaturated fatty acids changes which stimulated by RV, JEV, LPS, and Poly (I:C) for 24 h after treated by ST2825. (C-D) The saturated fatty acids and unsaturated fatty acids changes which stimulated by RV, JEV, LPS, and Poly (I:C) for 24 h after treated by PDTC.

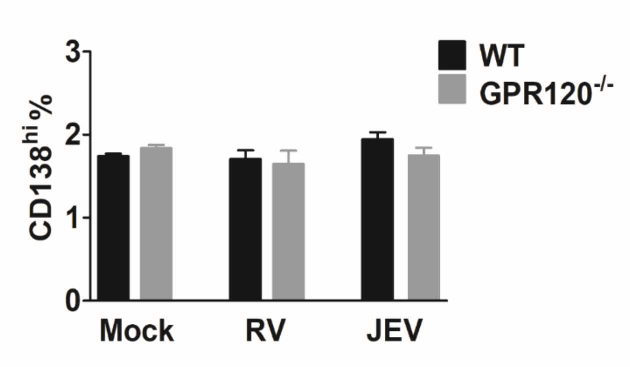

Figure S5. Flow-cytometric analysis of the proportion of the CD138 in CD11c positive cells of GPR120$^{-/-}$ mice after treated with RV, JEV. DC were collected from spleen of GPR120$^{-/-}$ mice after immunized by RV and JEV and stained with fluorescently labeled antibodies against CD11c and CD138.

Table S1. Primers used for quantification of FA transport proteins and FA ligand proteins mRNA.

Gene name	Sequence of forward primers (5'-3')	Sequence of reverse primers (5'-3')
Slc27a2	CAAGTCTCTGCTGCACTGCTTTC	GCCGACACTCCGTCTACTTTG
Slc27a4	TGCCCAGTCACCCAGACAAG	CATGCGGAATCCATAGTACACCAG
Fabp1	AGTGCGAACTGGAGACCATGAC	ACGGACTTTATGCCTTTGAAAGTTG
Fabp2	CACAGCTGAGATCATGGCATTC	CCATCCTGTGTGATTGTCAGTTTC
Fabp4	GCCTTTGTGGGAACCTGGAA	CCATTTACGCTGATGATCATGTTG
Fabp5	CTCCCACCATGGCCAGTCTTA	TGATGTTGTTGCCATCACACGTA
Prkaa2	CTGCTGGATGCCCAGATGAA	GAGATGACCTCAGGTGCTGCATAA
GPR40	GCTATTCCTGGGGTGTGTGT	CCCTGTGATGAGTCCTAACT
GPR43	GGCTTCTACAGCAGCATCTA	AAGCACACCAGGAAATTAAG
GPR84	TCCAATTCTGTCTCCATCCT	CTGACTGGCTCAGATGAAA
GPR120	CCATCCCTCTAGTGCTCGTC	TGCGGAAGAGTCGGTAGTCT
